# Genetic testing in psychiatry, the perceptions of healthcare workers and patients: a mini review

**DOI:** 10.3389/fpubh.2024.1466585

**Published:** 2024-10-10

**Authors:** Kyriakos I. Ioannou, Anastasia Constantinidou, Andreas Chatzittofis

**Affiliations:** ^1^Medical School, University of Cyprus, Nicosia, Cyprus; ^2^Department of Clinical Sciences and Psychiatry, Umeå University, Umeå, Sweden

**Keywords:** genetic testing, psychiatry, mental health, patients’ perspective, healthcare workers, precision medicine

## Abstract

**Background:**

Genetic testing in psychiatry has gained attention, raising questions about its application and impact. Understanding stakeholders’ perspectives, including healthcare providers and patients, is vital for informed policy development. The aim of this systematic review was to focus on the perceptions and concerns of patients and healthcare workers in psychiatry regarding the use of genetic testing.

**Methods:**

We conducted a systematic review following PRISMA guidelines, for the period 1/2/2014, to 1/1/2024, via PubMed and Embase databases identifying 50 articles in total. After excluding duplicates (*n* = 12), 38 articles went through screening. After careful full-text article assessment for eligibility and applying the inclusion and exclusion criteria, only fifteen (*n* = 15) of the articles were included.

**Results:**

Among 15 selected studies involving 3,156 participants (2,347 healthcare professionals; 809 patients), thematic analysis identified four primary themes: Organizational-implementation concerns, Ethical Considerations, Concerns on changes in clinical praxis, and Legal implications. Despite these concerns, seven out of eleven studies indicated that healthcare workers viewed genetic testing in psychiatry positively. Patients’ perspectives varied, with two of the four studies reflecting positive attitudes. No pervasive negative sentiment was observed.

**Conclusion:**

Our review highlights the multidimensional perspectives of healthcare professionals and patients surrounding the application of genetic testing in psychiatry. These considerations need to be addressed to facilitate the implementation of genetic testing in clinical praxis in psychiatry. Further research is needed for validation of the results and to guide policies and clinicians in the integration of genetic testing into mental healthcare practice.

## Introduction

Modern medicine is constantly improving its tools and modernizing its methods to be able to provide patients with the most effective and personalized treatment and genetic testing is in the frontline of precision medicine. The Center for Disease Control and Prevention, defines “genetic testing” as the process of scanning a person’s genetic sequence in search of alterations (mutations, variants) in DNA that may have clinical relevance (1).

A variety of clinical conditions in different fields of medicine are associated with genetic abnormalities and genetic testing is gaining ground in clinical praxis in different specialties (2–4). Genetic testing helps to identify the genetic component of some disorders, can be used in diagnosis, to guide treatment and for genetic counseling of the patient’s relatives. Additionally, monitoring of genetic markers during treatment can guide clinicians adjust treatment plans according to one’s genetic variations (5).

Psychiatry is a promising field for the application of genetic testing as, in comparison with other medical specialties, is still well behind in identifying biomarkers for diagnosis, prognosis and treatment response. However, epidemiological and genetic studies have shown a strong association between certain genetic alterations and psychiatric diseases, with expanding research in genetic causality and familial inheritance models (6). Due to the high prevalence of psychiatric disorders attributable to genetic alterations, the medical community has increased its interest in researching the genetic etiology of these disorders through genetic testing and familial risk classification (7). Considering the major impact of psychiatric diseases on society, investigation from such a revolutionary perspective could be crucial (8–11).

Psychiatric genetic testing can be helpful in unraveling the pathophysiology of common mental disorders such as schizophrenia, depression, bipolar disorders, and autism spectrum disorder. For instance, in schizophrenia, genetic testing examines variations in genes impacting functions like brain development, neurotransmission, and immune homeostasis (12). Information derived from genetic testing can be used as predictors and thus enable early interventions and empower individuals to make informed decisions about their health. Overall, psychiatric genetic testing serves as a crucial advancement in addressing complex psychiatric disorders and enhancing personalized healthcare strategies (13).

In addition to Mendelian models, genes-diseases correlation, and prenatal screening for genotypic abnormalities, the application of pharmacogenomics is increasing. Pharmacogenomics can revolutionize drug prescribing through tailoring treatments to each genetic profile, optimizing efficacy and minimizing unfavorable outcomes (14). It can reduce the trial-and-error period of treatment and provide actionable information in special populations (15). Variations in metabolic enzymes, including cytochrome P450 and especially within the CYP2D6 enzyme can impact the metabolism of antipsychotics and antidepressants, affecting treatment response. The use of diagnostic genetic tests remains relatively rare in current clinical practice, largely due to the complex polygenic nature of psychiatric disorders, which complicates direct genetic diagnosis (16). In contrast, pharmacogenomic tests are used more often, can help tailor medication, with growing evidence suggesting that they can improve both the tolerability and effectiveness of treatment. This has been shown in randomized clinical trials, systematic reviews and meta-analyses especially in managing mood disorders and schizophrenia (17–24). Thus, this distinction between the two test types is crucial, as their roles in psychiatric practice differs significantly.

Although there is increasing utility of genetic testing in medicine, the existing literature on the perceptions of healthcare professionals and patients regarding this practice is still growing. Initial studies report skepticism from both the medical community and patients regarding the efficacy and utility of these tests, and ethical concerns have been raised related to privacy, security of patient data, confidentiality, economic impact and possible harms such as psychological impact, stigmatization and discrimination (25, 26). Preliminary studies reported that the type of genetic condition can influence the mental health of patients when genetic testing is applied, with neurodegenerating disorders such as Huntington’s disease having a profound impact (27).

At present, there is a lack of comprehensive studies on the perspectives of healthcare professionals and patients regarding the utilization of genetic tests in psychiatry. Given the stigma often associated with psychiatric disorders and the vulnerability of psychiatric patients, it is important to investigate the perspectives of those directly involved. Additional concerns might include the capacity of psychiatric patients and their relatives to consent to genetic testing and whether they understand the implications and possible benefits. Also, questions arise about the necessity of such tests in severely mentally ill patients and the usefulness of the results (28). Therefore, the identification of perceptions of healthcare professionals and patients has important implications in the application of genetic testing in psychiatric clinical praxis.

Thus, the aim of this systematic review was to identify, categorize and discuss the perceptions, opinions and concerns of patients and healthcare workers in psychiatry regarding the use of genetic testing.

## Materials and methods

The systematic review was based on the Preferred Reporting Items for Systematic reviews and Meta-Analysis statement (Prisma) guidelines (29).

### Data sources and searches

The research of the medical literature was executed in PubMed and Embase and was confined to the preceding decade encompassing the timeframe of 1/12014 to 1/1/2024. The selection of this research timeframe was based on the recognition that while genetic testing in psychiatric applications had been apparent earlier, more advanced applications including pharmacogenetics, were developed in recent years (30). Published articles were collected using a standard search strategy search query: (“Genetic testing” OR “Genetic screening” OR “Molecular diagnostics”) AND (“Psychiatry” OR “Mental health” OR “Psychiatric disorders” OR “Mental illness”) AND (“Perceptions” OR “Attitudes” OR “Beliefs” OR “Opinions”) AND (“Healthcare workers” OR “Clinicians” OR “Healthcare professionals” OR “Medical staff”) AND (“Patients” OR “Individuals” OR “Participants” OR “Subjects”).

### Study selection

This systematic overview encompassed articles that investigated the attitudes, perspectives, beliefs, and views of healthcare providers and patients concerning genetic testing in psychiatry. Research articles on Alzheimer’s disease (*n* = 2) were excluded due to the primary characteristics of this disease including memory and cognitive dysfunctions. Νo other exclusion criteria referring to gender, age, or ethnicity have been applied. Only articles written in English were considered for inclusion. The PRISMA 2009 checklist guided any similarly restrictions that were imposed on the articles.

### Data extraction, study quality assessment and analysis

The systematic search resulted in the identification of 50 articles in total. Out of the 50 articles, 12 were excluded as duplicates resulting in 38 articles for further screening. After careful full-text article assessment for eligibility and applying the inclusion and exclusion criteria, only 20 (*n* = 20) of the articles were deemed eligible to be further assessed in full study. Three (*n* = 3) articles were excluded as identified as a review while two (*n* = 2) more articles were excluded as being out of the scope of this systematic review since they did not include opinions or beliefs for genetic testing in psychiatry but in another specialties. All the references of the identified review articles were also checked for eligibility. Thus, finally, 15 studies reporting on the patients’ and healthcare provider’s perspectives regarding genetic testing in psychiatry were included. The Prisma Flowchart for exclusion/inclusion criteria is presented in Supplementary Figure S1.

All included studies were thematically analyzed for identifying themes related to genetic testing in psychiatry. The thematic analysis was performed separately on healthcare professionals and patients’ opinion/perspectives regarding the topic. Data were retrieved from each study and classified through thematic analysis first in subthemes and subsequently into the different themes.

## Results

### Studies characteristics

The studies included (*n* = 15) had a total population of 3,156 participants including 2,347 healthcare professionals and 809 patients. The studies were carried out in diverse locations, most common the United States (*n* = 8). The mental healthcare professionals included were psychiatrists, psychiatric pharmacists, child and adolescent psychiatrists, psychiatric genetics researchers and general practitioners working in mental health clinics. The patients included in the studies suffered from depression, bipolar disorder, schizophrenia, and patients receiving treatment for a mental health condition. The studies are shown in Table 1.

**Table 1:** Studies including perspectives of mental health providers (*n* = 2,347) and patients’ perspectives.

Studies	Year	Study type	Sample size	Type of healthcare providers	Study design	Country
Aboelbaha et al. (39)	2023	Qualitative	18	Psychiatrists	Semi-structured online interviews	Qatar, Kuwait, Oman, Saudi Arabia, United Arab Emirates (UAE), Bahrain, Egypt, Jordan, Iraq, Palestine, Tunisia, and Algeria.
Chan et al. (40)	2017	Quantitative	194	Psychiatrists (167) and psychiatric pharmacists (27)	Anonymous web-based questionnaire	Singapore
Kostick et al. (41)	2017	Qualitative	39	Psychiatric genetics researchers	Semi-structured online interviews	United States, Australia, Canada, Denmark, Germany, Asia, and other European countries.
Laplace et al. (42)	2021	Quantitative	397	Psychiatrists and psychiatry residents	Online self-administrated questionnaire	France
Shisko et al. (43)	2015	Quantitative	91	Psychiatric pharmacists	Cross sectional survey through a third party website	United States, Indonesia, Singapore, Abu Dhabi, and Canada
Soda et al. (44)	2023	Quantitative	958	Child and adolescent psychiatrists	Cross-sectional design survey	United States
Thompson et al. (45)	2015	Quantitative	113	Psychiatrists	Cross-sectional design survey	United States
Undurraga et al. (46)	2021	Quantitative	123	Psychiatrists	Cross-sectional design survey via e-mail	Chile
Vest et al. (47)	2020	Qualitative	31	Psychiatrists and general practitioners	Focus groups	United States
Walden et al. (48)	2015	Quantitative	168	Psychiatrists and general practitioners	Pharmacogenetics in psychiatry Follow-up” (PIP-FQ) questionnaire via email	Canada
Wolfe et al. (49)	2018	Quantitative	215	Child psychiatrists (121) and intellectual disability psychiatrists (94)	Cross sectional online survey	United Kingdom
Kastrinos et al. (50)	2020	Quantitative	598	Psychiatric patients who had received treatment for mental health conditions	Cross-sectional survey—online questionnaire	United States
Liko et al. (51)	2020	Qualitative	20	Patients with major depressive disorder or bipolar disorder with depressive symptoms	Semi-structured interviews	United States
McCarthy et al. (52)	2020	Quantitative	170	Patients were veterans receiving care for depression	Cross sectional online survey	United States
Cullen et al. (53)	2020	Qualitative	21	Individuals with schizophrenia and first-degree relatives of people with schizophrenia	Semi-structured telephone interviews	Australia

### Healthcare professionals’ perspectives on genetic testing in psychiatry

Four themes and fifteen subthemes were identified, the most common theme was Organizational-Implementation concerns, including six subthemes: cost, limited knowledge and training, lack of clear guidelines, lack of funding, limited availability and accessibility, and lack of collaborations among clinicians and researchers. The second theme, Ethical Considerations, encompasses subthemes such as informed consent, patient-doctor relationship, psychological distress, stigma, and inappropriate treatment decisions or changes. The third theme, Concerns on changes in clinical praxis, includes three subthemes: threat to clinical expertise, accuracy of tests, and managing patient expectations. The final theme, Legal Implications, includes concerns related to employment.

All themes, subthemes, and studies identifying them are shown in Table 2.

**Table 2:** Identified concerns of healthcare providers in psychiatry regarding genetic testing.

Primary concerns of healthcare providers	Subthemes	Studies
Organizational-implementation concerns	Cost	(39, 40, 42–46, 51)
	Limited knowledge and training	(39–49)
	Lack of clear guidelines	(39–41, 44)
	Lack of funding	(43, 49)
	Limited availability and accessibility	(46, 48, 49)
	Lack of collaborations of clinicians and researchers	(41, 45)
Ethical considerations	Informed consent	(39, 48, 49)
	Patient-doctor relationship	(39, 42)
	Psychological distress and insurability	(40, 42, 43, 45, 47)
	Stigma	(40, 41, 45, 48, 49)
	Inappropriate treatment decisions or changes (Misuse of samples)	(44, 45, 49)
Concerns on changes in clinical praxis	Threat to clinical expertise	(39)
	Accuracy of tests	(40, 51, 44, 46, 47)
	Managing patient expectations	(51)
Legal implications	Concerns related to employment	(40)

### Patients’ perspectives on genetic testing in psychiatry

Four main categories, with a total of 11 subcategories were identified. These primary categories mirror those identified in the case of healthcare professionals including organizational-implementation concerns, ethical considerations, implications for clinical practice, and legal implications. A more comprehensive overview of the themes observed in each individual study is presented in Supplementary Table S1.

### What was the general sentiment among healthcare providers and patients?

Out of the eleven (*n* = 11) studies where mental health providers were involved, seven (*n* = 7) studies suggested that the consensus among psychiatry professionals was that genetic testing is a valuable tool in psychiatry. However, in four (*n* = 4) studies, mental health professionals have expressed mixed perspectives on the matter.

Regarding the patients’ perspective, positive opinions regarding genetic tests in psychiatry were expressed, in two (*n* = 2) studies, while in two (*n* = 2) studies, patients had mixed perspectives. It is important to note that among the studies included in this systematic review, there was no indication of a general negative opinion regarding genetic tests among healthcare providers in psychiatry and psychiatric patients.

## Discussion

To our knowledge, this is the first systematic review to investigate the perspectives of patients and healthcare workers associated with genetic testing in psychiatry with the findings contributing to a deeper understanding of the utilization of genetic testing in psychiatry and shed light on ethical considerations and potential risks from various perspectives. The theme that was most frequently identified was the concerns expressed by professionals regarding the limited education, training, and professional experience on genetic testing and genetic information. Indeed, although genetic testing and counseling have many applications in medicine, are still not included as part of the fundamental curriculum in the majority of medical schools across the world (31). Consequently, this leads to a dearth of professional education and specific training in clinical settings that directly impacts the perspectives of healthcare providers with regards to the utilization of these technologies and techniques in their daily practice. Thus, it is imperative that practitioners receive adequate training to be able to fully utilize newer technologies in clinical praxis such as pharmacogenetic testing (32).

The concern ranking second in frequency among professionals was the cost associated with genetic testing. The cost was deemed a prominent barrier hindering the implementation of pharmacogenomics testing. Physicians expressed concerns regarding the affordability of these tests suggesting that enhancing accessibility and affordability, potentially through the public health sector, would be of utmost importance (33). Given the novelty and innovative nature of genetic counseling applications, it becomes evident that the public health sectors are ill-prepared to handle the demand and coverage for such tests, resulting in an unfortunate situation where patients bear the economic burden and their perspective on genetic testing becomes adversely affected. Especially in the field of Psychiatry with vulnerable patients of often poor socioeconomic status and services underfunded, it is crucial to advocate for cost coverage of new technology. Nevertheless, in order for genetic testing to truly become a routine practice in the field of medicine and to be fully covered by insurance companies or public health sectors, it is imperative that it provides the scientific community with more clear and consistent results, as well as a wider range of applications.

One of the most prevalent concerns among healthcare providers related to the potential mental health stigma experienced by their patients, which, if left unaddressed, could result in discrimination and self-stigmatization for individuals identified as having risk variants. To mitigate the potential negative consequences on participants and their families, clinicians who integrate genetic counseling into their daily practice must actively consider strategies for alleviating such impacts. Furthermore, an extended educational approach targeting both patients and the general public could play a pivotal role in eradicating the stigmatization endured by these patients, as the limited knowledge surrounding genetics has been identified as the primary factor influencing such behavior (34). By equipping individuals with a comprehensive understanding of the genetic components underlying mental health, it becomes possible to combat the stigma associated with it, fostering an environment of inclusivity and support. This comprehensive approach ensures that patients receive the necessary care without facing unnecessary barriers or prejudice stemming from a lack of awareness or understanding.

Special concerns were raised regarding the potential psychological impact of genetic testing in psychiatry. Indeed, it was previously reported that mental health professionals expressed concerns regarding the possible influence of genetic testing on the mental well-being of patients (28). The characteristics of psychiatric disorders affecting the cognitive function of patients, together with the stigma of psychiatric disorders has the risk for increased misunderstandings and negative psychological impact from genetic testing results. In addition, patients may undergo distress regarding their condition, alterations in treatment, or anxiety regarding possible consequences such as employability. Such concerns, subsequently, tend to overshadow the consideration of the delicate equilibrium associated with the “right to know.” As previous studies have suggested, clinicians are confronted with the task of meticulously evaluating the patients’ entitlement to access their genetic information against the potential psychosocial detriments that may emerge from receiving certain outcomes, including heightened stress, anxiety, stigma, and discrimination (14). However, it is reasonable to mention that, to the best of our knowledge, there is currently no evidence supporting a high risk of psychological harm related to genetic testing among psychiatric patients, and previous studies suggest that the severity of psychological risks posed by genetic testing is not substantial (35, 36). At this juncture, further research is necessary to identify specific patient groups, such as individuals with mental health conditions, who may have a heightened susceptibility to psychological harm. It is possible that some patients might assert that knowledge of genetics and genetic testing could dampen the hopes of individuals afflicted with mental disorders. The awareness of patients regarding their genetic predisposition could potentially engender a sense of fatalism or hopelessness regarding their condition.

Alongside the possible psychological impact of genetic testing results, the uncertainty surrounding the outcomes of the tests is also important. Certain patients express worries regarding the efficacy of genetic testing in the event of inconclusive or non-actionable results. In such cases, patients believe that genetic testing would offer limited benefits. It is widely acknowledged that not all genes that can predict the response to psychiatric treatment have been discovered (16). While there is strong evidence and available guidelines for genes associated with drug metabolism, such as CYP2C19 and CYP2D6, as well as other pharmacokinetic implications, the evidence for other genes is still limited (37). This lack of comprehensive identification of genes can potentially hinder the effectiveness of pharmacogenetic testing and raise concerns within the medical community regarding the accuracy and applicability of such tests.

In the context of the two aforementioned concerns, clinicians have a valid and justifiable concern about effectively managing patient expectations. It is imperative to emphasize that pharmacogenetic testing serves as a clinical decision-making instrument utilized by healthcare providers alongside other pertinent factors to facilitate the process of medication selection. It is crucial for healthcare providers to adeptly handle patients’ expectations in terms of acknowledging the limitations of pharmacogenetic testing as well as the varying degrees of evidence associated with the genes being tested (38).

Considering the unique concerns of professionals, psychiatrists expressed concerns regarding an excessive reliance on genetic tests, as it may undermine their clinical expertise and experience. There were concerns that this reliance may replace clinical judgment and impact the patient-doctor rapport, ultimately leading to the dehumanization of the therapeutic alliance and psychiatric care. Furthermore, psychiatrists also expressed worries about the feasibility of incorporating discussions about testing and patient education within the time constraints of routine care visits. Conversely, some professionals believed that genetic testing could streamline the process by identifying the most appropriate medication for each patient, potentially reducing the trial-and-error approach. These varying perspectives among professionals highlight concerns surrounding the actual utility and potentials of genetic counseling.

This study has several limitations. A key limitation of this review is the use of the vote-counting method, which treats all studies equally, regardless of differences in sample size, quality, or bias control. This approach can oversimplify findings, as it does not account for the weight or statistical significance of individual studies, potentially leading to skewed conclusions. Given these limitations, the findings presented should be interpreted cautiously. Future research would benefit from qualitative or meta-analytic approaches to better capture the complexity and variation across studies on genetic testing in psychiatry. As the literature search was conducted in PubMed and Embase only in the English language, possible bias cannot be excluded. Also, the timeframe of the study, including the last decade, might have resulted in missing earlier studies in the subject. However, genetic testing in psychiatry has mainly been used the most recent years. Also, a bias toward healthcare professionals in psychiatry from developed countries, especially USA was evident. As there was a large variation in the methodology of the included studies, including interviews, focus groups, questionnaires and surveys in different patient populations and settings, a meta-analysis was not possible. Finally, as a selection bias cannot be excluded, in combination with the small number of studies, with some themes derived from a small number of studies, the results should be viewed with caution. Although within the studies, the review reached thematic saturation, further research might illuminate new aspects contributing new themes. The relatively emerging nature of this field may have contributed to the lack of research on this topic, thereby limiting the range of insights that could be obtained from existing literature. In this review diagnostic and pharmacogenomic tests were grouped together as a separate analysis was not feasible due to lack of data and data heterogeneity. While both are forms of genetic testing, diagnostic genetic testing is rarely employed due to the complexity of psychiatric disorders, while pharmacogenomic testing has an increasing role in clinical praxis (17–24). Future research should focus more specifically on the evolving evidence surrounding pharmacogenomic testing, which holds greater relevance to current psychiatric practice. Despite these limitations, this study provides valuable insights into the current landscape of attitudes and perceptions toward genetic testing in psychiatry, highlighting the need for further research to address these gaps in understanding.

In conclusion, this review systematically examined the perceptions of healthcare providers and patients regarding genetic testing in psychiatry highlighting concerns, including the limited knowledge, training and the high costs of genetic tests. This study also emphasizes the importance of establishing clear and updated guidelines to minimize errors, improve test accuracy, and enhance patient confidence in genetic testing. To achieve these goals, increased funding and further investigation into this subject are imperative.
