# Supplementary material for: Genetic testing in psychiatry, the perceptions of healthcare workers and patients: a mini review
Source: Front Public Health. 2024 Oct 10;12:1466585. doi: 10.3389/fpubh.2024.1466585 (PMC11499203; doi:10.3389/fpubh.2024.1466585)
Supplement: Supplementary file 1 [file Data_Sheet_1.docx]

Figure S1. Prisma Flowchart for the systematic review
