# Supplementary material for: Genetic testing in psychiatry, the perceptions of healthcare workers and patients: a mini review
Source: Front Public Health. 2024 Oct 10;12:1466585. doi: 10.3389/fpubh.2024.1466585 (PMC11499203; doi:10.3389/fpubh.2024.1466585)
Supplement: Supplementary file 2 [file Table_1.docx]

**Table S1.** Identified Concerns of Psychiatric patients in genetic testing.

| **Primary Concerns of Patients** | **Subthemes** | **Studies** |
| --- | --- | --- |
| **Organizational-implementation concerns** | Cost | 51 |
| **Ethical Considerations** | Uncertainty of the Patients | 50, 53 |
|  | Psychological distress | 52, 53 |
|  | Stigma | 52 |
|  | Patient Expectations | 52, 53 |
|  | Accuracy of tests | 53 |
| **Concerns on changes in clinical praxis** | Safety | 50 |
|  | Actionable findings | 50, 53 |
|  | Inappropriate treatment decisions or changes (Misuse of samples) | 53 |
| **Legal Implications** | Privacy and Confidentiality | 52 |
|  | Concerns related to employment | 53 |
